# Analysis of intact prophages in genomes of *Paenibacillus larvae*: An important pathogen for bees

**DOI:** 10.3389/fmicb.2022.903861

**Published:** 2022-07-15

**Authors:** Henrique G. Ribeiro, Anna Nilsson, Luís D. R. Melo, Ana Oliveira

**Affiliations:** ^1^LIBRO – Laboratório de Investigação em Biofilmes Rosário Oliveira, Centre of Biological Engineering, University of Minho, Braga, Portugal; ^2^LABBELS – Associate Laboratory on Biotechnology and Bioengineering, and Electromechanical Systems, Centre of Biological Engineering, University of Minho, Braga, Portugal; ^3^Department of Ecology, Swedish University of Agricultural Sciences, Uppsala, Sweden

**Keywords:** *Paenibacillus larvae*, prophage, bacterial evolution, bacterial fitness, bacterial virulence

## Abstract

*Paenibacillus larvae* is the etiological agent of American Foulbrood (AFB), a highly contagious and worldwide spread bacterial disease that affects honeybee brood. In this study, all complete *P. larvae* genomes available on the NCBI database were analyzed in order to detect presence of prophages using the PHASTER software. A total of 55 intact prophages were identified in 11 *P. larvae* genomes (5.0 ± 2.3 per genome) and were further investigated for the presence of genes encoding relevant traits related to *P. larvae*. A closer look at the prophage genomes revealed the presence of several putative genes such as metabolic and antimicrobial resistance genes, toxins or bacteriocins, potentially influencing host performance. Some of the coding DNA sequences (CDS) were present in all ERIC-genotypes, while others were only found in a specific genotype. While CDS encoding toxins and antitoxins such as HicB and MazE were found in prophages of all bacterial genotypes, others, from the same category, were provided by prophages particularly to ERIC I (enhancin-like toxin), ERIC II (antitoxin SocA) and ERIC V strains (subunit of Panton-Valentine leukocidin system (PVL) LukF-PV). This is the first in-depth analysis of *P. larvae* prophages. It provides better knowledge on their impact in the evolution of virulence and fitness of *P. larvae*, by discovering new features assigned by the viruses.

## Introduction

As the more abundant entities on Earth, bacteriophages (or phages), are considered prime performers in the dynamics of bacterial populations. Phages are generally categorized into two, groups based on their lifecycle: virulent phages (strictly lytic) and temperate phages (lysogenic). The first group have an exclusively lytic lifestyle, always resulting in lysis of the host cell after infection. In the lysogenic cycle, the phage integrates the host genome becoming a prophage and it can remain at this stage for several bacterial generations (Fortier and Sekulovic, 2013). If external stimuli occur, causing bacterial stress, prophages may be excised from the bacterial chromosome and follow the lytic cycle.

It is widely recognized that temperate phages, capable of interacting with the host genome, are major contributors to the diversity and evolution of most bacterial communities in all ecosystems. The prophage-host interactions are a result of coevolution processes (Koskella and Brockhurst, 2014; Harrison and Brockhurst, 2017; Olszak et al., 2017; Khan et al., 2020). On sharing genes, prophages play a key role in modulating bacterial ability to infect their host, to compete with other bacteria and cause disease (virulence), or to adjust metabolism according to environmental conditions in order to survive and grow (fitness). On preventing superinfection events, prophages support the lysogenic state of their hosts and ensure the propagation of their progeny (Bobay et al., 2014; Fortier, 2017).

Prophage inputs of new genes into the host can be achieved either by its vertical propagation on bacterial lines or by transduction (horizontal gene transfer, HGT), i.e., when fragments of bacterial DNA are wrongly packed inside phage capsids and then propagated among infected bacteria. For example, prophages can influence traits such as resistance to starvation, biofilm formation, antibiotic tolerance or improved toxicity (Fortier and Sekulovic, 2013; Touchon et al., 2017; Costa et al., 2018). Several reports have revealed that the presence of prophages can increase the virulence and the toxicity of a bacterial host in many ways. For example, nonvirulent strains of *Escherichia coli*, *Vibrio cholerae*, and *Clostridium botulinum* have become virulent by acquiring prophages with toxin genes (Shiga toxin, Cholera toxin, and Botulinum toxin, respectively; Barksdale and Arden, 1974; O’Brien et al., 1984; Waldor and Mekalanos, 1996). Further, *Streptococcus mitis* holds adhesion factors encoded by tail genes carried by prophages and *Salmonella enterica* gained enzymes such as superoxide dismutase and neuraminidase, which improves the antioxidant ability and their virulence (Bensing et al., 2001; Figueroa-Bossi et al., 2001; Feiner et al., 2015). Prophages can protect the lysogenic bacteria against further infections by similar phages and confer an advantage against competing non-lysogenic bacteria by hampering or delaying their colonization through prophage induction (Brussow et al., 2004).

Enzymes such as integrases, recombinases or excisionases combine homologous DNA sequences between temperate phage and bacteria genome (Lewis, 2001; Feiner et al., 2015). This mechanism can occur randomly in the host genome, a strategy used for example by the Mu phage, or at specific and conserved locations in the genome, such as for the *λ* phage (Bondy-Denomy and Davidson, 2014; Toussaint and Rice, 2017).

In nature, the continuous presence of a prophage genome in a bacteria often leads to degradation of genetic sequences, a phenomenon called “phage domestication,” also known as Muller’s ratchet (Touchon et al., 2014). The host genome seems to inactivate the newly integrated phages and then get rid of undesirable genes by means of genetic degradation (point mutations and deletions) on genetic regions not under selection (Bobay et al., 2014). This mechanism can justify why most prophage sequences usually found in bacterial genomes are incomplete and do not contain essential genes for phage-host interaction (e.g., integrases, endolysins) or lack genes coding for essential structural proteins (Casjens, 2003; Bobay et al., 2014; Czajkowski, 2019). The rates of genetic decay rates seem to be dependent on bacterial robustness, but, for example, in *E. coli*, they have been described as slow (Bobay et al., 2014).

*Paenibacillus larvae* is a spore-forming Gram-positive bacterium that causes the most severe bacterial honeybee brood disease, American Foulbrood (AFB; Genersch, 2010). AFB is associated with great economical losses in apiculture, as current legislation does not allow European beekeepers to use antibiotics (European Parliament and the Council of the European Union, 2010) and in many European countries it is mandatory to burn all colonies showing disease signs. The severity of AFB varies with the *P. larvae* genotype involved in the infection (Genersch et al., 2005). Five different genotypes, ERIC-types (Enterobacterial Repetitive Intergenic Consensus), have been identified for *P. larvae* so far: ERIC I and II are frequently found in AFB outbreaks; ERIC III and IV have lower epidemiological relevance as they are rarely found; ERIC V is a recently isolated and identified genotype (Rauch et al., 2009; Beims et al., 2020). The pathogenesis of *P. larvae* varies between each genotype and depends on the functional toxins genes and secondary metabolites of the genotypes (Müller et al., 2015; Ebeling et al., 2016; Genersch, 2017).

Although phages have been proposed to be valuable solutions for mitigation of AFB. All 50 *P. larvae* phages reported to date are temperate (Oliveira et al., 2013; Beims et al., 2015, 2020; Carson et al., 2015; Tsourkas et al., 2015; Abraham et al., 2016; Merrill et al., 2018; Walker et al., 2018; Yost et al., 2018; Ribeiro et al., 2019), and, to our knowledge, there are no studies analyzing how these phage genomes cause impact on the host phenotype.

In this study, prophage-like sequences found in all complete *P. larvae* genomes available at GenBank (NCBI) were identified and analyzed at the genomic level. The prevalence of such sequences in *P. larvae* genomes and the contribution of the prophages for the evolution of *P. larvae* virulence and fitness are herein explored, as far as the five ERIC genotypes are concerned.

## Materials and methods

### Data collection

All *P. larvae* genomes deposited on GenBank until April 2020 (a total of 14 chromosomes and 20 plasmids) were analyzed (minimum genome coverage of 50x) and named from H1 to H14 (Table 1). Prophage regions were named from R1 to Rn, placed after the reference of the respective host.

**Table 1:** *Paenibacillus larvae* strains and respective reference name, accession number, genome sequencing method and coverage, genotype classification, GC content, genome size, number of contigs, and respective sizes.

Host	Strain	Reference strain	Accession no.	Sequencing method	Genome coverage	ERIC genotype	GC%	Size (Mbp)	No. of contigs	Size range of contigs (Kbp)	Prophages (validated)		
											Total	Intact	Defective
3*	DSM 25719	DSM 25719	NZ_ADFW00000000.1	Sanger dideoxy sequencing; 454; Illumina	94	I	44.1	4.58	8	8.1–3,664	21	8	13
5^a^	MEX14		NZ_LAWY00000000	454	50	I	44.0	4.19	139	0.5–213.6	17	3	14
6	ATCC 9545	ATCC 9545	NZ_CP019687.1	PacBio	147.4	I	44.2	4.29	NA	NA	13	5	8
7	ATCC 13537	ATCC 13537	NZ_CP019794.1	PacBio	56.4	IV	44.3	4.41	NA	NA	16	3	13
8	CCM 38	CCUG 7429	NZ_CP020327.1	PacBio	150.8	IV	44.3	4.33	NA	NA	15	5	10
9*	SAG 10367	SAG 10367	NZ_CP020557.1	PacBio	214.2	II	44.1	4.67	NA	NA	18	7	11
10	ERIC_I	DSM 7030	NZ_CP019651.1	PacBio; Illumina HiSeq2500	193	I	44.2	4.29	NA	NA	15	5	10
11*	ERIC_III	LMG 16252	NZ_CP019655.1	PacBio; Illumina HiSeq2500	114	III	44.2	4.49	NA	NA	18	7	11
12	ERIC_IV	LMG 16247	NZ_CP019659.1	PacBio; Illumina HiSeq2500	113	IV	44.3	4.27	NA	NA	15	3	12
13	DSM 25430; ERIC_II	DSM 25430	NZ_CP019652.1	PacBio; Illumina HiSeq2500	153	II	45.0	4.02	NA	NA	12	1	11
14*	ERIC_V	DSM 106052	CP019717.1	PacBio; Illumina HiSeq2500	257	V	44.1	4.67	NA	NA	21	8	13
						Average GC content	44.3			Total validated	181	55	126
**Host (Excluded)**	**Strain**	**Reference strain**	**Accession no.**	**Sequencing method**	**Genome coverage**	**ERIC genotype**	**GC%**	**Size (Mbp)**	**No. of contigs**	**Size range of contigs (Kbp)**	**Prophages (excluded)**		
											**Total**	**Intact**	**Defective**
1^b^	BRL-230010		NZ_AARF00000000.1	454	50	I	44.1	3.98	646	0.25–58.6	23	0	23
2^b^	B-3650	LMG 16245	NZ_ADZY00000000.3	Sanger; Illumina	1; 100	I	44.1	4.35	353	0.05–331.7	9	0	9
4^c^	DSM 25430	DSM 25430	NC_023134.1	Sanger dideoxy sequencing; 454; Illumina	64	II	45	4.05	NA	NA	8	0	8

The last three columns refer to the number of total, intact and defective prophages present in each strain, after manual validation. *Hosts with genome size >4.49 Mbp and with ≥7 no. of intact prophages. The second table details data from strains excluded from the analysis.

a Host 5 (MEX14) classified as ERIC I without experimental validation; homology and position in the ERIC I branch of phylogenetic tree available on NCBI database.

b The high number of contigs available restricted an accurate analysis.

c Sequence reported by Djukic et al. (2014), identical to the obtained latter (re-sequenced) by Beims et al. (2020). NA, not applicable.

### Detection of prophages in *Paenibacillus larvae* strains

Prophage sequences were obtained until April 2020, for each of the *P. larvae* accession numbers, using PHASTER (PHAge Search Tool Enhanced Release) webserver^1^ (Zhou et al., 2011; Arndt et al., 2016; Table 1). PHASTER output distinguished intact, questionable and incomplete phage genomes, depending on the number of coding DNA sequences (CDS) of a region attributable to prophages, and on the presence of phage-related genes. Here, questionable and incomplete prophages were both denominated “defective.” After the identification by PHASTER, prophages were manually cured for increased accuracy. In cases where important elements for phage infection were missing, such as the N-acetylmuramoyl-l-alanine amidase (an endolysin, the most conserved gene present in *P. larvae* phages; Stamereilers et al., 2018), other genes with lysis function, structural genes (e.g., major capsid, tail, tail fiber), holins or DNA packaging genes (small and large terminase subunits), these were not considered as intact prophages.

### Identification of potential virulence factors encoded by prophages

BLASTp was used to assess phage coding sequences (CDS) functions, using default parameters and against tailed phages (tax id: 28883), simultaneously, and Conserved Domains-Search Tool (in Pfam database with E-value cut-off of 1 × 10^−5^; Finn et al., 2014). Complete genomes were checked for antibiotic resistance genes through the Resistance Gene Identifier (RGI) of The Comprehensive Antibiotic Resistance Database (CARD), under the “perfect, strict and loose hits” criteria^2^ (Alcock et al., 2020).

To assist with prophage curing and classification, the proteins were grouped into seven functional categories: virion structure, virion assembly, host lysis, DNA replication/metabolism, gene regulation, host virulence/fitness functions and lysogeny/transduction (Supplementary Tables S1 and S2; Xia and Wolz, 2014; Stamereilers et al., 2018).

An adapted Cluster of Orthologous Groups (COG) of proteins were generated by comparing the protein sequences and grouped according to the function. The specific functions of host virulence and fitness-related proteins were detailed in new categories, as well included the category of unknown functions. It also maintained the six previous categories used in prophages proteins classification.

Special attention was given to CDS with functions that could somehow have influenced host evolution such as those that allow phage lysogeny or transduction and those capable of modulating host virulence or fitness.

### Taxonomic classification of prophages

The phage taxonomic family was attributed according to the presence of characteristic structural proteins. For the *Siphoviridae* family (non-contractile tail phages) a set of four proteins close to each other should be present [major tail protein (MTP); two tail assembly proteins (TAP); and tail tape measure protein (TMP); Pell et al., 2009; Mahony et al., 2016]. The presence of the tail sheath protein (TSP) was enough to suggest that prophages belong to the *Myoviridae* family (phages with contractile tail; Pell et al., 2009; Veesler and Cambillau, 2011; Aksyuk et al., 2012; Mahony et al., 2016), this assumption was also strengthened when three other proteins tail tube protein (TTP); one tail assembly protein (TAP) and tail tape measure protein (TMP), were present close to the TSP and tail fiber proteins. Phages lacking the MTP, TMP, TTP or TSP were classified as *Podoviridae* (phages with non-contractile short tail; Veesler and Cambillau, 2011; Aksyuk et al., 2012). To support the previous classification, each prophage genome was compared with the GenBank database using BLASTn. In addition, the PHASTER classification presented the phage with the highest number of proteins similar to the one analyzed.

### Whole-genome comparison

The phage genome alignments and the phylogenetic tree were constructed by the MAFFT algorithm (Katoh and Standley, 2013) and Geneious Tree Builder, using the Neighbor-Joining method with bootstrapping of 100 and Tamura-Nei genetic distance model, respectively, present in Geneious R9 (Biomatters, Newark, NJ, United States). All the previously reported *P. larvae* phages (*n* = 50; Supplementary Table S3) and all *P. larvae* intact prophages (*n* = 55) identified here were included in the analysis (Supplementary Table S4). The identity matrix of the phylogenetic trees generated was used to infer on whole identity. Clusters were defined whenever different phages shared at least 60% nucleotide identity and subclusters if the identity was higher than 90% (Stamereilers et al., 2018; Oliveira et al., 2019). In case of less than 60% identity with any other phage, it was treated as a singleton.

### Statistical analysis

The statistical analysis of the results was performed using GraphPad Prism 7 (GraphPad Software, San Diego, CA, United States). Results were compared using one-way ANOVA, with Turkey’s multiples comparison statistical test, in the prevalence and average of each class (total, intact or defective), two-way ANOVA, with Turkey’s multiples comparison statistical test, comparisons between both classes of prophages (intact vs. defective) and multiple comparisons between the different genotypes for each class of prophages (intact vs. defective). For GC content comparison between prophages versus host, the unpaired Welch’s *t*-test was used. All tests were performed with a confidence level of 95%. Differences were considered statistically significant if value of *p* ≤ 0.05.

## Results

### Prevalence of prophage sequences in *Paenibacillus larvae* complete genomes

**Figure 1:**
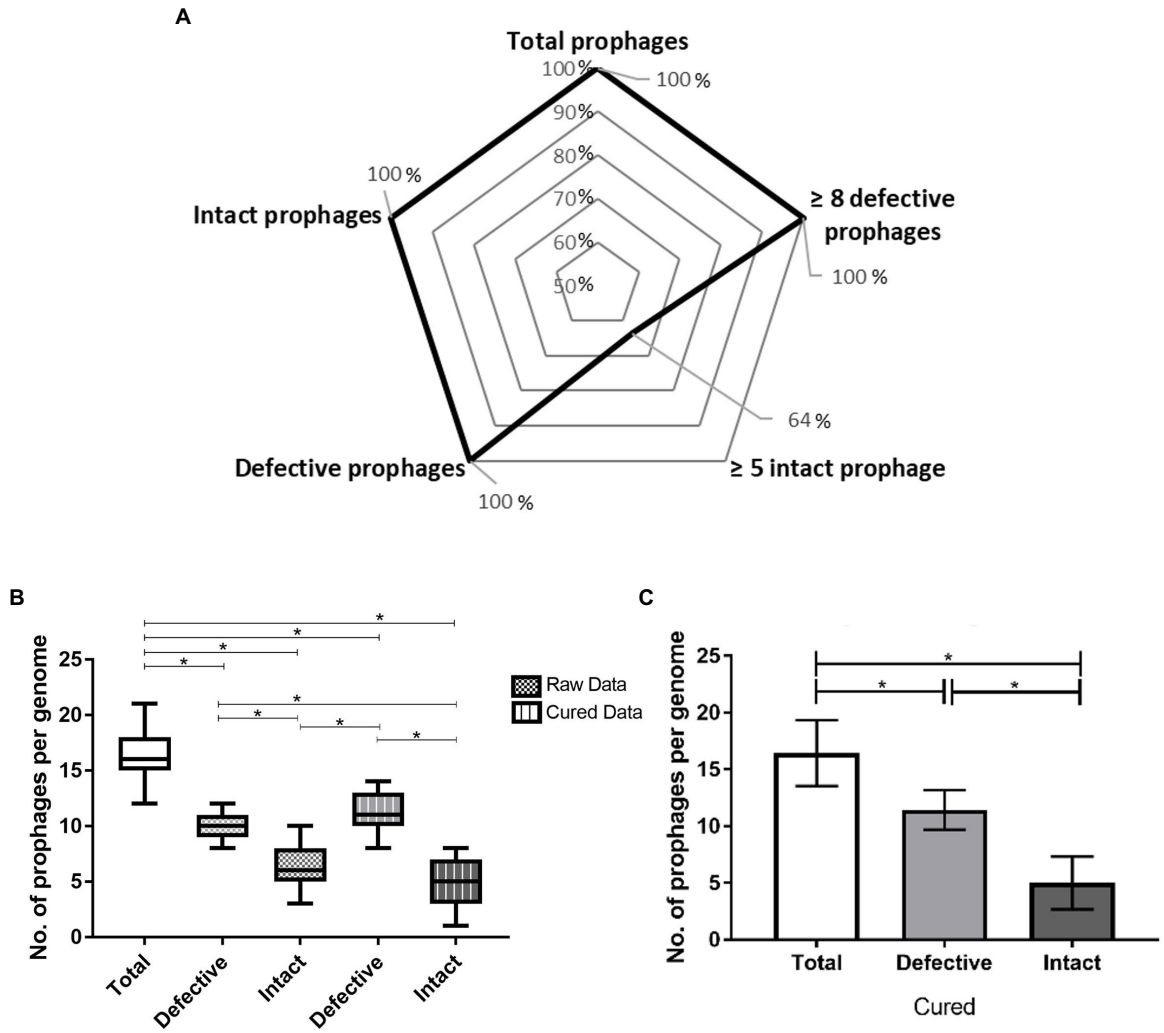
Prophage prevalence in *Paenibacillus larvae* genomes: **(A)** Percentage of hosts with ≥ one and ≥ five intact prophages and ≥ one and ≥ eight defective prophages. **(B)** Whisker plots of prophage frequency per bacterial genome (total, defective and intact) before and after manual curing. Raw data provided directly from PHASTER, cured data results from manual verification. The horizontal line of each box represents the average prophages per genome and the external edges to the minimum/maximum number. **(C)** Average of total, defective, and intact prophages present per host genome. The error bars indicate the SD. Statistically significant, if value of *p* < 0.05 (*).

Despite 14 *P. larvae* genomes being available in the GenBank (NCBI), only 11 were analyzed [Host 1 and 2 were both excluded due to genome fragmentation and low-confidence results; Host 4 and 13 had the same genomic sequence, and therefore, only one of them was considered (Host 13, the most recently reported)] and in all of them prophage-like elements were identified (Table 1; Figure 1A). From a total of 181 prophage-related sequences (174 in chromosomes and seven in plasmids), 71 were intact (70 in chromosomes and one in a plasmid) and 110 were defective prophages (104 in chromosomes and six in plasmids; Table 1). However, the manual curing of these sequences only confirmed 55 intact prophages (all in chromosomes) and consequently included 16 more defective prophages (15 in chromosome and one in a plasmid; Figure 1B). All *P. larvae* genomes harbored at least one intact prophage. The average was 5.0 ± 2.3 prophages per genome (Figure 1C), varying in size between 23.6 and 108.1 kbp (Supplementary Table S4). The average GC content of prophages and *P. larvae* genomes was 43.5% ± 2.6 (Supplementary Table S4) and 44.3% ± 0.3 (Table 1), respectively, and the former occupied 5.83% ± 2.45 of the latter, [variation between 1.76% (Host 13) and 9.71% (Host 3; Supplementary Table S4)].

**Figure 2:**
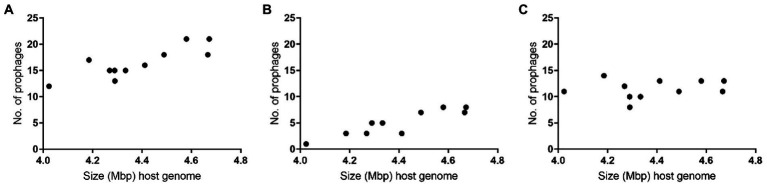
Number of prophages per size of host *Paenibacillus larvae* genomes: **(A)** total **(B)** intact **(C)** defective.

Overall, the larger the *P. larvae* genome, the higher the number of intact prophages were observed: Host 3, 9, 11 and 14, the four largest bacterial genomes (>4.49 Mbp), actually included between seven and eight prophages, while Host 5, 6, 7, 8, 10, 12 and 13, smaller, harbored less than five (Table 1; Figure 2).

**Figure 3:**
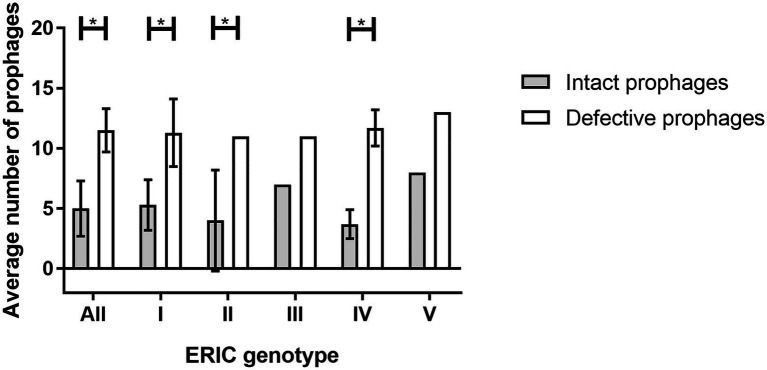
Average number of prophages (intact and defective) present per ERIC genotype (ERIC I-V). The error bars indicate the SD. Statistically significant, if value of *p* < 0.05 (*).

Comparatively intact, the defective prophages were always present in higher number (Table 1), and at least eight sequences (average of 11.5 ± 1.8) were identified per genome for all the hosts analyzed (Figures 1A,C). No differences between the number of intact or defective prophages per ERIC genotype (*p* > 0.05) was observed (Figure 3).

This work mainly focused on the analysis of intact prophages, considering that these have a more direct impact on the spreading of new traits to their hosts by completing their lytic cycle.

### Prophage protein library

**Figure 4:**
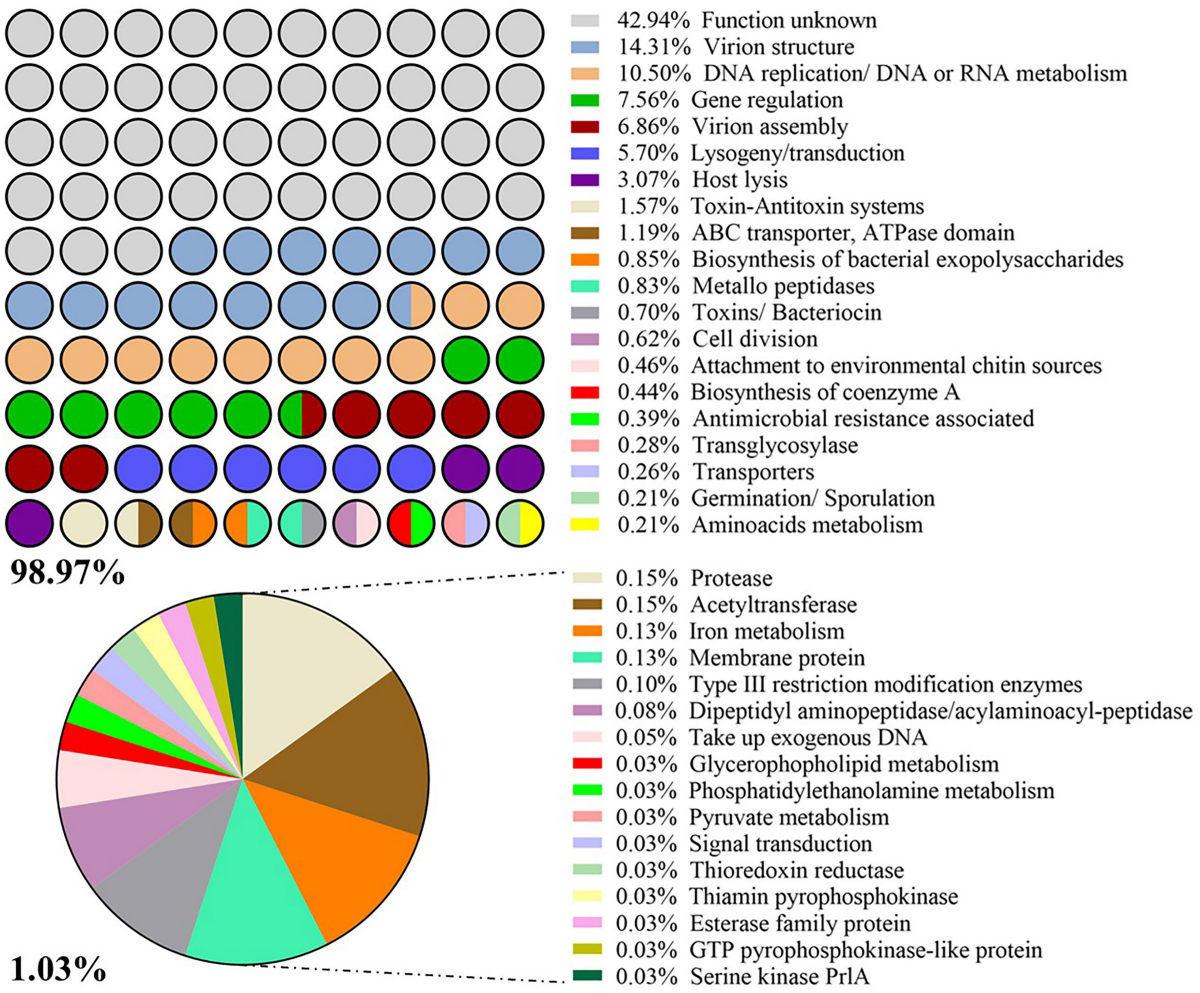
List of categories Cluster of Orthologous Groups (COG). Frequency (%) of prophage-derived CDS with a given function per COG.

A total of 3,876 CDS were identified among the 55 intact prophage genomes. All CDS were grouped into 36 functional categories using COG. Around 43% of the groups encoded proteins with unknown function. The frequency of CDS per COG is illustrated in Figure 4.

The role of the prophage genes was not experimentally confirmed, and therefore, the analysis was conducted relying on the homologies provided by their amino acid (aa) sequences, using BLASTp. In average, each protein had 194 ± 162 aa. The largest, with 1,234 aa, was identified as the tail tape measure protein (TMP) and was present in H3_R14, H10_R4 and H10_R10, and the shortest, 28 aa, a HP present in H6_R8, H10_R4, H11_R15 and H12_R13.

About 95% of *P. larvae* prophage CDS have at least one homologous sequence with tailed phages (tax id: 28883; based on NCBI non-redundant database). Associated with prophage ability to transport and exchange genomic DNA fragments between hosts, transposases seemed to be the most frequently present enzymes in prophages (found 112 CDS encoding them), followed by 39 integrases and 20 recombinases. Together with some regulators, these three enzymes represented 5.7% in the COG analysis (Figure 4). A set of 68 phage CDS, globally related to antimicrobial resistance, toxicity for bacteria/larvae [toxin-antitoxin (TA) systems, toxins] or transport of substances, metabolism and germination/sporulation events were subsequently identified as having potential influence on host performance (Table 2; Supplementary Table S5). Due to the high diversity of host functions associated with virulence and fitness, the percentage of each individual trait was low less than 2% in the COG analysis. TA systems was the category with the highest percentage (1.6%; Figure 4).

**Table 2:** Coding DNA sequences (CDS) identified in prophages potentially influencing host virulence or fitness.

	ERIC I				ERIC II		ERIC III	ERIC IV			ERIC V
CDS	Host 3	Host 5	Host 6	Host 10	Host 9	Host 13	Host 11	Host 7	Host 8	Host 12	Host 14
TetR family transcriptional regulator (TetR/AcrR)											
β-Lactamase superfamily domain [metal β-lactamases (MBL) fold metallo-hydrolase Yycl]											
β-Lactamase inhibitory proteins (BLIP)											
ATP-binding cassette (ABC) group											
Aromatic acid exporter family protein											*
Major facilitator superfamily (MFS) transporter											
Multidrug efflux small multidrug resistance (SMR) transporter											
Efflux transporter-like protein		*									
Iron–sulfur (Fe-S) cluster assembly protein SufB								*		*	
NifU family iron–sulfur (Fe-S) cluster assembly protein—SUF system								*		*	
Metal β-lactamases (MBL) fold metallo-hydrolase											
Phosphomannomutase											
Transglycosylase											
Toxin HicA											
Antitoxin HicB											
Antitoxin MazE											
Antitoxin SocA					*						
AbrB/MazE/SpoVT family DNA-binding domain-containing protein											
Bacterial toxin 44											*
Toxin											
Toxin-like protein											*
Bacteriocin biosynthesis protein		*									
Epsilon-toxin type B (EtxB)											
Leukocidin LukF-PV precursor = leukotoxin											
Closticin	*										
DNA internalization-related competence protein ComEC/Rec2		*	*								
Segregation and condensation protein B (ScpB)											
YopX family protein											
Enhancin / phosphohydrolase		*									
Virulence-associated protein E											
ImmA/IrrE family metallo-endopeptidase											
Esterase family protein		*									
S8 family serine peptidase											
Histidine kinase-like protein		*									
Pyruvate dehydrogenase E1 component subunit alpha		*									
Metallopeptidase											
FtsX-like permease family protein					*						
GlcNAc-chitin binding protein (GbpA)											*
Dipeptidyl aminopeptidase/acylaminoacyl-peptidase											
GTP pyrophosphokinase-like protein	*										
Host-nuclease inhibitor protein Gam (Gam)											
MazG-like nucleotide pyrophosphohydrolase					*						
VRR-NUC domain-containing protein											
YifB family Mg chelatase-like AAA ATPase		*	*	*							
YncE family protein							*				
YxeA family protein											
Coat protein											
Iron-containing alcohol dehydrogenase		*									
Ketopantoate hydroxymethyltransferase											
Membrane protein											*
Outer spore coat protein (CotE)						*					
Spore protease YyaC	*	*	*	*							
Sporulation protein YhbH	*										
Stress protein	*	*	*	*							
DNA mismatch repair protein MutS						*					
ERF superfamily protein											
Acetyltransferase											
dCMP deaminase family protein		*	*								
Murein transglycosylase-like protein		*									
NTP-binding protein	*										
Peptidase domain				*							
Phenylalanine racemase											
Phosphatidylglycerophosphatase A (PgpA)							*				
Phosphatidylserine decarboxylase		*									
STAS-like domain-containing protein		*									
Thiamin pyrophosphokinase		*									
Thioredoxin reductase											*
YqaE/Pmp3 family membrane protein											

The gray color identifies the corresponding CDS in the respective ERIC genotype. *CDS only found in this genotype.

Although RGI analysis did not indicate any functional antimicrobial resistance (AMR) gene, AMR-related sequences, such as TetR family transcriptional regulator of a tetracycline resistance mechanism, the β-lactamase superfamily domain (MBL fold metallo-hydrolase Yycl) that hydrolyses the β-lactam antibiotics class B or the β-lactamase inhibitory proteins (BLIP), able to inhibit a variety of class A β-lactamases such as the penicillin antibiotics were identified. Few CDS seemed to be also involved in the transport of antibiotics out of bacterial cells [e.g., multidrug efflux small multidrug resistance (SMR) transporter], a mechanism associated with antimicrobial resistance.

There were CDS for other types of transporter proteins, either generic, such as ATP-binding cassette (ABC), the major facilitator superfamily (MFS), efflux, small multidrug resistance (SMR) or very specialized ones- aromatic acid exporter and iron–sulfur (Fe-S) cluster assembly proteins SufB and NifU.

The analyzed prophages also harbored TA systems. For example, for the *hicAB* system, consisting of the HicA toxin and HicB antitoxin, both parts were identified, while for *mazEF* or *socAB* systems, only the antitoxin part of the TA cassette was present.

Prophages further possess CDS that putatively confer virulence traits against bee larvae. These include metallopeptidases like enhancin, *Yersinia* outer proteins (Yops) like YopX, a N-acetylglucosamine (GlcNAc)-chitin binding protein (GbpA), the precursor of a subunit of Panton-Valentine leukocidin system (PVL) LukF-PV, a pore -forming epsilon-toxin type B (EtxB), a bacteriocin-like closticin and the DNA internalization competence protein ComEC/Rec2.

The research found CDS for enzymes that may interfere with host metabolism and regulation, such as phosphomannomutases, transglycosylases, a pyruvate dehydrogenase E1 and the histidine kinase-like protein. Finally, our analysis suggested the presence of CDS that can be involved in sporulation and germination, like the outer spore coat protein CotE, sporulation protein YhbH, and spore protease YyaC.

### Distribution of proteins related to putative host traits according to ERIC genotype

Despite the low number of available genomes representing each of the *P. larvae* ERIC genotypes (Table 1), prophages with proteins involved in bacterial fitness (metabolic functions, transport of nutrients, sporulation and germination) or virulence (like toxins, bacteriocins and AMR-related proteins) were identified in all ERIC genotypes, and some were exclusive to a given genotype (Table 2). For example, in ERIC I strains, 21 unique proteins were observed, including transporters (an efflux transporter and the DNA internalization protein ComEC/Rec2), a bacteriocin (closticin and enhancin-like protein), enzymes (histidine kinase and pyruvate dehydrogenase E1) and sporulation or germination-related proteins, while in ERIC II strains, the antitoxin SocA, the FtsX-like permease, the MazG-like nucleotide pyrophosphohydrolase, the structural protein involved in spore formation, CotE, and the DNA mismatch repair protein MutS were exclusively present. The proteins identified only in ERIC III strains were YncE, related with DNA binding, and PgpA, a phosphatidylglycerophosphatase, in ERIC IV were involved in iron–sulfur (Fe-S) uptake (SufB) and nitrogen fixation (NifU) and in ERIC V were proteins from the aromatic acid exporter family, a leukocidin subunit LukF-PV precursor, two other toxins, a membrane protein and the chitin-binding protein GbpA.

However, some prophage-derived proteins were found to be shared between genotypes. For example, the pore-forming toxin EtxB and the host-nuclease inhibitor protein Gam, were identified in ERIC I and V strains. ERIC I, III and IV strains share a protein participating in chromosomal partition during cell division (segregation and condensation protein B, ScpB), and other responsible for the racemization of phenylalanine (phenylalanine racemase). Virulence-associated protein E was only identified in ERIC I and II strains, while ERIC I, II and V share a S8 family serine peptidase and an acetyltransferase. The β-lactamase inhibitory protein (BLIP) and a coat protein were present in both ERIC III and IV strains. From the transporter proteins previously enumerated, the MFS transporter was only present in ERIC I and III strains, while the multidrug efflux SMR transporter was present in ERIC I, III and IV strains. Proteins such as phosphomannomutase, HicB and MazE antitoxins or YopX family protein were identified in all ERIC genotypes, while the transcriptional regulator of the TetR family, the HicA toxin, the transglycosylase and the ImmA/IrrE were present in all except the recently reported ERIC V.

### Prophage taxonomy

All new phage genomes analyzed encode the TMP, and therefore no podoviruses were identified. Table 3 details the structural proteins in the base of prophage morphology assumptions and subsequent taxonomic classification. Based on the defined criteria, 34 of the 55 prophages were assigned as siphoviruses. Of these 34, four genomes contain all genes encoding structural proteins that distinguish this taxonomic group, 13 did not have one of the proteins (TAP or MTP/TTP) and 17 were described as *Siphoviridae* members. The latter, despite not having both TAP and MTP/TTP also miss the exclusive protein TSP of the *Myoviridae* family. This classification, supported by BLASTn and PHASTER analysis, revealed high homology (*E*-value = 0; Coverage between 29% and 94%, Identity >88.03%) with other previously reported *P. larvae Siphoviridae* phages (Stamereilers et al., 2018).

**Table 3:** Taxonomic classification of prophages based on structural proteins present (Y: protein present).

Prophage	TSP	MTP/TTP	TAP	TAP	TMP	Family	Most common phage (PHASTER)	BLASTn parameters			
								Homolog phage	Coverage (%)	Identity (%)	*E*-value
H3_R2					Y	*Siphoviridae* ^ *a* ^	Vegas (*P. larvae*)	Dragolir	67	99,92	0
H3_R3		Y^#^	Y	Y	Y	*Siphoviridae*	Tripp (*P. larvae*)	Heath	38	90,61	0
H3_R5		Y^#^	Y	Y	Y	*Siphoviridae*	Fern (*P. larvae*)	Jacopo	87	99,15	0
H3_R6			Y	Y	Y	***Siphoviridae***	Tripp (*P. larvae*)	Tripp	75	99,45	0
H3_R11	Y	Y^#^	Y		Y	*Myoviridae*	Lily (*P. larvae*)	Lily	41	84,28	0
H3_R14		Y^#^	Y		Y	***Siphoiridae***	Vegas (*P. larvae*)	Hayley	72	99,98	0
H3_R15	Y	Y^*^	Y	HP	Y	Myoviridae^*^	Jimmer1 (*B. laterosporus*)	Yerffej	11	99,37	0
H3_R18		Y^#^			Y	*Siphoviridae* ^ *a* ^	Vegas (*P. larvae*)	Vegas	29	99,47	0
H5_R2	Y	Y^*^	Y	HP	Y	*Myoviridae*	Harrison (*P. larvae*)	Harrison	25	99,97	0
H5_R6		Y^#^			Y	*Siphoviridae* ^ *a* ^	Vegas (*P. larvae*)	Hayley	32	99,47	0
H5_R13		Y^#^	Y	Y	Y	*Siphoviridae*	Fern (*P. larvae*)	Leyra	38	97,91	0
H6_R1		Y^#^			Y	*Siphoviridae* ^ *a* ^	Vegas (*P. larvae*)	Vegas	29	99,47	0
H6_R3		Y^#^	Y		Y	***Siphoviridae***	Harrison (*P. larvae*)	Paisley	96	99,98	0
H6_R6			Y	Y	Y	***Siphoviridae***	Tripp (*P. larvae*)	Heath	61	90,57	0
H6_R7	Y	Y^*^	Y		Y	*Myoviridae* ^*^	Jimmer1 (*B. laterosporus*)	Harrison	9	96,25	0
H6_R8		Y^#^	Y		Y	***Siphoviridae***	Vegas (*P. larvae*)	Hayley	72	99,98	0
H7_R1			Y		Y	*Siphoviridae* ^ *a* ^	Tripp (*P. larvae*)	Scottie	94	99,91	0
H7_R6		Y^#^			Y	*Siphoviridae* ^ *a* ^	Vegas (*P. larvae*)	Dragolir	36	96,08	0
H7_R10		Y^#^	Y	Y	Y	*Siphoviridae*	Rani (*P. larvae*)	Diva	18	84,37	0
H8_R2	Y	Y^*^	Y		Y	*Myoviridae* ^*^	Jimmer1 (*B. laterosporus*)	Harrison	11	94,73	0
H8_R6	Y	Y^*^	Y	HP	Y	*Myoviridae* ^*^	Abouo (*B. laterosporus*)	Dragolir	22	93,18	0
H8_R7	Y	Y^#^	Y		Y	*Myoviridae*	Lily (*P. larvae*)	Lily	79	91,09	0
H8_R8		Y^#^			Y	*Siphoviridae* ^ *a* ^	Vegas (*P. larvae*)	Dragolir	36	96,09	0
H8_R12			Y		Y	*Siphoviridae* ^ *a* ^	Tripp (*P. larvae*)	Scottie	94	99,97	0
H9_R3	Y	Y^*^	Y		Y	*Myoviridae* ^*^	Jimmer1 (*B. laterosporus*)	Wanderer	11	89,43	0
H9_R4	Y	Y^*^	Y		Y	*Myoviridae* ^*^	Jimmer1 (*B. laterosporus*)	Paisley	9	86,88	0
H9_R5		Y^#^	Y		Y	***Siphoviridae***	Vegas (*P. larvae*)	Diane	41	92,1	0
H9_R8			Y	Y	Y	***Siphoviridae***	Tripp (*P. larvae*)	C7Cdelta	66	91,32	0
H9_R10			Y	Y	Y	***Siphoviridae***	Tripp (*P. larvae*)	Ley	55	89,51	0
H9_R14	Y	Y^*^	Y		Y	*Myoviridae* ^*^	Jimmer1 (*B. laterosporus*)	Sitara	15	86,09	0
H9_R15	Y	Y^*^	Y		Y	*Myoviridae* ^*^	Jimmer1 (*B. laterosporus*)	Leyra	7	97,86	0
H10_R4		Y^#^	Y		Y	***Siphoviridae***	Vegas (*P. larvae*)	Vadim	99	99,95	0
H10_R5	Y	Y^*^	Y		Y	*Myoviridae* ^*^	Jimmer1 (*B. laterosporus*)	Yerffej	11	99,37	0
H10_R7			Y	Y	Y	***Siphoviridae***	Tripp (*P. larvae*)	Heath	74	90,61	0
H10_R10		Y^#^	Y		Y	***Siphoviridae***	Harrison (*P. larvae*)	Paisley	96	99,99	0
H10_R12		Y^#^			Y	*Siphoviridae* ^ *a* ^	Vegas (*P. larvae*)	Hayley	34	99,46	0
H11_R3		Y^#^	Y		Y	***Siphoviridae***	Fern (*P. larvae*)	Likha	18	85,1	0
H11_R8			Y		Y	*Siphoviridae* ^ *a* ^	Tripp (*P. larvae*)	Scottie	94	99,98	0
H11_R10	Y	Y^*^	Y		Y	*Myoviridae* ^*^	Jimmer1 (*B. laterosporus*)	Harrison	9	94,91	0
H11_R12	Y	Y^*^	Y		Y	*Myoviridae* ^*^	Abouo (*B. laterosporus*)	Harrison	11	94,73	0
H11_R13	Y	Y^*^	Y	HP	Y	*Myoviridae* ^*^	Abouo (*B. laterosporus*)	Dragolir	23	93,18	0
H11_R14	Y	Y^#^	Y		Y	*Myoviridae*	Lily (*P. larvae*)	Lily	79	91,09	0
H11_R15		Y^#^			Y	*Siphoviridae* ^ *a* ^	Vegas (*P. larvae*)	Dragolir	36	96,08	0
H12_R3		Y^#^	Y		Y	***Siphoviridae***	Fern (*P. larvae*)	Yerffej	19	84,49	0
H12_R8			Y		Y	*Siphoviridae* ^ *a* ^	Tripp (*P. larvae*)	Scottie	94	99,98	0
H12_R13		Y^#^			Y	*Siphoviridae* ^ *a* ^	Vegas (*P. larvae*)	Dragolir	36	96,09	0
H13_R11			Y		Y	*Siphoviridae* ^ *a* ^	Tripp (*P. larvae*)	Scottie	60	88,03	0
H14_R2			Y		Y	*Siphoviridae* ^ *a* ^	Tripp (*P. larvae*)	Tripp	52	89,95	0
H14_R3	Y	Y^#^	Y		Y	*Myoviridae*	Lily (*P. larvae*)	phiERICV	94	100	0
H14_R7	Y	Y^*^	Y	HP	Y	*Myoviridae* ^*^	Jimmer2 (*B. laterosporus*)	Sitara	11	87,86	0
H14_R8	Y	Y^*^	Y	HP	Y	*Myoviridae* ^*^	Jimmer2 (*B. laterosporus*)	Paisley	17	86,26	0
H14_R9			Y		Y	*Siphoviridae* ^ *a* ^	Tripp (*P. larvae*)	Heath	67	89,07	0
H14_R10	Y	Y^*^	Y	HP	Y	*Myoviridae* ^*^	Jimmer1 (*B. laterosporus*)	phiERICV	7	94,99	0
H14_R14			Y		Y	*Siphoviridae* ^ *a* ^	Tripp (*P. larvae*)	Heath	76	89,11	0
H14_R15	Y	Y^*^	Y	HP	Y	*Myoviridae*	Harrison (*P. larvae*)	phiERICV	12	95,37	0

TSP, tail sheath protein; MTP, major tail tube protein; TTP, tail tube protein; TAP, tail assembly protein; TMP, tail tape measure protein; Y^#^, one of both proteins present; Y^*^, only TTP present; HP, hypothetical protein; *Siphoviridae*^*a*^—prophages from this family based on phage homology of BLASTn; *Siphoviridae*—prophages with the four typical proteins; ***Siphoviridae***—three of the four typical proteins. Myoviridae^*^—prophages homologous to *Brevibacillus laterosporus* phages; *Myoviridae*—prophages homologous to other siphovirus phages.

The remaining 21 prophage genomes, when containing genes encoding the TSP were considered to belong to the *Myoviridae* family. Genome comparison revealed that, while 15 prophages were genetically close to members from the same family, (*Brevibacillus laterosporus* phages Jimmer1, Jimmer2 and Abouo; Sheflo et al., 2013), the remaining six shared identity (84%–100%, with a coverage between 12% and 94%) with previously described siphoviruses, such as phage Lily.

### Whole-genome comparison of intact prophages

**Figure 5:**
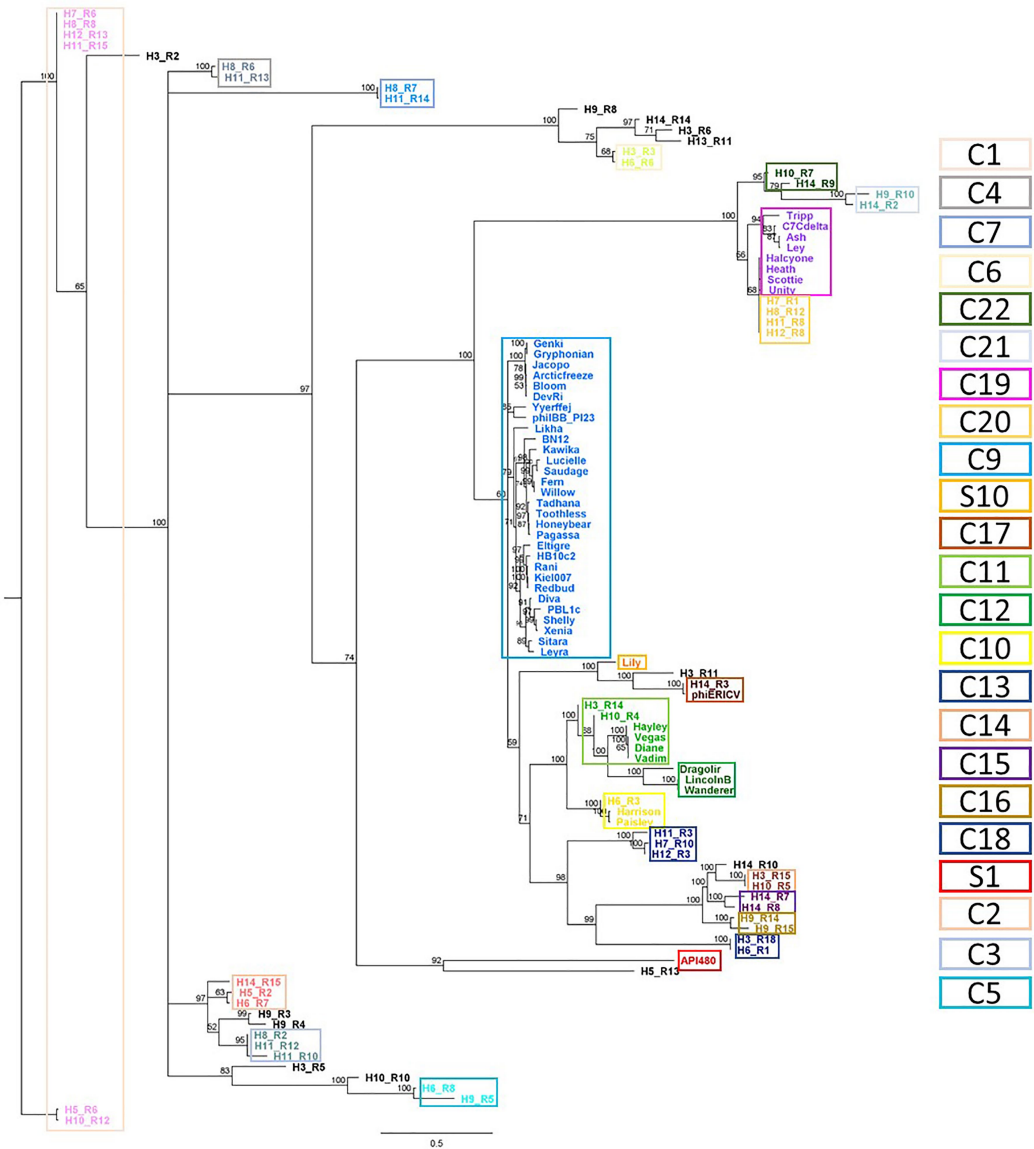
Phylogenetic analysis of *Paenibacillus larvae* phages. Whole genomes based on shared CDS content (nucleotide), obtained with Geneious. Database: *Paenibacillus larvae* reported phages (*n* = 50) and intact *Paenibacillus larvae* prophages (*n* = 55), identified here. Clusters have ≥60% of shared CDS and were highlighted by colored rectangles.

A previous analysis, grouped *P. larvae* phages into four clusters (Fern, Halcyone, Harrison and Vegas) and two singletons (Lily and API480; Ribeiro et al., 2019). The re-alignment included these newly identified intact prophages revealed 12 singletons (here S1 to S12) and 22 clusters (here C1 to C22), the latter divided into 51 subclusters (from A to AY; Figure 5; Supplementary Table S6).

This comparison changes the identity between phage genomes adjusts two of the previously reported groups (Ribeiro et al., 2019). The introduction of prophages H3_R4 and H10_R4 resulted in a division of the Vegas cluster into two new clusters, C11 (including H3_R4 and H10_R4) and C12. Besides, comparatively to Harrison, C10 has one more phage, H6_R3. The remaining new clusters or singletons do not introduce changes to the previously reported by Ribeiro et al. (2019): C9, C19, S1 and S10 fully correspond to Fern, Halcyone, API480 and Lily, respectively.

In most cases, the same cluster comprised prophages from different hosts. The exceptions were C3, C15 and C16, where prophages H11_R10 and H11_R12, H14_R7 and H14_R8 and H9_R14 and H9_R15 share the Host 11 (with 83.8% identity), 14 (with 71.4% identity) and 9 (with 61.4% identity), respectively (Figure 5; Supplementary Table S6). Similar phages were found in different hosts, as for example, H11_R15 and H12_R13 (cluster C1–C), H11_R8 and H12_R8 (cluster C20–AU) or H11_R14 and H8_R7 (cluster C7–J).

## Discussion

Temperate phages can remain in a dormant state within their host without triggering the lytic cycle, while at the same time having a considerable impact on the host genome variability and evolution, modulating the host fitness and virulence (Brussow et al., 2004; Harrison and Brockhurst, 2017). To our knowledge, there has been no attempts to explore the role of temperate phages in the ecology and evolution of *P. larvae* despite studies reporting a total of 51 *P. larvae* prophages in the last 8 years (Oliveira et al., 2013; Beims et al., 2015, 2020; Carson et al., 2015; Tsourkas et al., 2015; Abraham et al., 2016; Merrill et al., 2018; Walker et al., 2018; Yost et al., 2018; Ribeiro et al., 2019; Bozdeveci et al., 2021).

Here, 11 complete genomes of *P. larvae* previously isolated from AFB outbreaks (Heyndrickx et al., 1996; Alippi et al., 2002; Genersch et al., 2005; Djukic et al., 2014; Peréz de la Rosa et al., 2015; Beims et al., 2020) were analyzed to identify the presence of intact prophages. In total, 55 full-length *P. larvae* phage genomes were identified and analyzed *in silico* and their potential to influence forthcoming generations by providing new features was investigated.

Prophages were identified in the *P. larvae* genomes using both the software PHASTER and by manual curing. The manual curing indicated an incorrect estimate of prophages (both intact and defective) in the software analyses (Table 1). Despite PHASTER is a commonly used software for prophage prediction, some inaccuracies, are being described (Arndt et al., 2019).

Surprisingly, the set of *P. larvae* plasmids identified did not hold any intact prophage, contrarily to what has been reported for other species, such as *Acinetobacter baumannii* (Costa et al., 2018). Only defective prophages were found in the *P. larvae* plasmids (Supplementary Table S2). A similar phenomenon was observed in *Clostridium difficile* DLL3026 plasmids, with some defective prophages encoding structural and integrase genes (Amy et al., 2018).

There was no apparent association between ERIC genotype and the number of prophages per genome. On average, each *P. larvae* strain harbored five intact prophages. The presence of multiple intact prophages in a single strain (poly-lysogenic strains) has been reported for both Gram-positive and Gram-negative bacteria (Touchon et al., 2016; Garriss and Henriques-Normark, 2020). In Gram-positive bacteria, up to five prophages have been observed in a single *C. difficile* genome (Fortier, 2018), around two prophages per genome have been reported for *Bacillus thuringiensis* (Fu et al., 2019) and *Lactococcus lactis* (Ruiz-cruz et al., 2020), and in *S. aureus* four prophages have been observed (Bae et al., 2006). In Gram-negative bacteria, more than two prophages per genome have been reported for *Citrobacter rodentium* and *Klebsiella pneumoniae* (Magaziner et al., 2019; Bleriot et al., 2020) and for enterotoxigenic *E. coli* more than eight prophages per genome (Wang et al., 2020). Because of the superinfection exclusion mechanism that prevents infection by similar phages, a poly-lysogenic strain may become less susceptible to accepting new prophages (Touchon et al., 2016).

As expected, the presented data suggest a positive correlation between the number of integrated prophages and the size of the genome of bacteria. If on the one hand, the presence of so many intact prophages increases the length of the host genome, on the other hand, larger genomes provide higher stability to hold prophages (Touchon et al., 2016; Costa et al., 2018). From an evolutionary point of view, it is possible that hosts harboring more prophages do not benefit from the integration of new prophages and consequently will not accept genes providing novel advantageous functions.

The comparison between the whole genomes of the newly identified and the previously described *P. larvae* phages (Oliveira et al., 2013; Beims et al., 2015, 2020; Carson et al., 2015; Tsourkas et al., 2015; Abraham et al., 2016; Merrill et al., 2018; Walker et al., 2018; Yost et al., 2018; Ribeiro et al., 2019) proposes changes in some of the existent clusters (Vegas and Harrison; Ribeiro et al., 2019) and introduces new ones (more 18 clusters and 10 singletons). Before this study, four clusters (Fern, Harrison, Vegas, Halcyone) and two singletons, (API480 and Lily) had been reported (Ribeiro et al., 2019) and recently, Bozdeveci et al. (2021) introduced a new phage similar to API480, proposing a new cluster. This new data brings new insights into the diversity of *P. larvae* phages and supports the importance of a permanent feed and update of the available phage genomic data.

Besides genomics, the present analysis also contributes to widening the taxonomic diversity of *P. larvae* phages. So far, most of the reported phages are siphoviruses (Oliveira et al., 2013; Stamereilers et al., 2018; Beims et al., 2020), with the exception of two podoviruses (Ribeiro et al., 2019; Bozdeveci et al., 2021). The present analysis suggests the inclusion of at least 15 myoviruses in the collection. Furthermore, this suggests a discussion on the classification of phage Lily and homologous as myoviruses. Concerning Lily phage, it is of note that, besides being reported as a member of the *Siphoviridae* family, Lilly encodes proteins typically found in myoviruses such as TSP, TMP and one TAP (gp15), the tail tube protein (gp14), tail fiber proteins (Stamereilers et al., 2018) and lacks the MTP gene. This might introduce some ambiguity in comparisons with such phage.

Our prophage analysis further disclosed a set of proteins that can putatively influence host fitness and pathogenesis. Despite the lower incidence of CDS originating these categories (Figure 4), they seem to be the most impacting on the host phenotypic transformation. For example, the small multidrug resistance (SMR) transporter, found in three of the analyzed prophages, seems to provide the cell the ability to escape antibiotics by transporting drugs out of the cell. Partial sequences of AMR (e.g., genes encoding inhibitory proteins of the β-lactam antibiotics class A and B) or CDS with regulatory functions concerning the tetracycline resistance mechanism were apparently observed, but contrarily what has already been reported for other bacteria (Stanczak-Mrozek et al., 2017; Wachino et al., 2019), none of the analyzed prophages are able to provide the host a functional AMR. They might be a result of previous attempts to use antibiotics to control AFB in hives (Nakajima et al., 1997; Reybroeck et al., 2012).

Our analysis also suggests the presence of proteins involved in the transport of either generic [ATP-binding cassette (ABC), MFS transporter, efflux transporter] or specific (aromatic acid exporter, Fe-S cluster transporter) substances. The bioavailability of iron has been shown to be recognizably vital for *P. larvae* growth (Hertlein et al., 2014). This makes the identification of proteins involved in iron uptake (SufB and NifU protein) in two prophages particularly interesting, as they may provide the host with important fitness advantages. Although the YncE protein is of unknown function it has been assumed in the past that was related to iron metabolism (McHugh et al., 2003), but a more recent characterization showed that it is associated with DNA-binding activities (Kagawa et al., 2011).

Prophage genomes also harbor proteins with homology to others associated with *P. larvae* metabolism and regulation. Usually, the histidine kinase enzyme plays role in signal transduction across the cellular membrane by phosphotransfer and phosphatase activity. Here, a histidine kinase-like protein was identified in one prophage, and perhaps this could induce the host to phosphorylate the response regulator *agrC* associated with a common quorum sensing system, something that was previously reported in a *C. difficile* prophage (Hargreaves et al., 2014a,b; Taylor et al., 2019). The presence of ComEC/Rec2, a protein enabling DNA internalization was identified in two prophages and can confer the ability for uptake of exogenous DNA from the environment, promoting the HGT (Solomon and Grossman, 1996). The enzyme phosphomannomutase was identified in several of the analyzed prophages. This enzyme may play a role in several functions involving biofilm formation (biosynthesis of bacterial exopolysaccharides), protection against environmental factors and the actions of antibiotics (Regni et al., 2002).

Although it has been reported that prophages may strongly impact bacterial virulence by providing new toxins through lysogenic conversion, as described for botulism toxin in *C. botulinum*, Shiga toxin in *E. coli* or Cholera toxin in *V. cholerae* (Barksdale and Arden, 1974; O’Brien et al., 1984; Waldor and Mekalanos, 1996; Feiner et al., 2015), the present suggests that some toxins encoded by *P. larvae* prophages may only influence the strain itself through the presence of TA systems rather than affecting bee larvae. The activity of TA systems usually leads to the inhibition of cell growth by interfering with several cellular processes. Biologically, their functions are generally associated with growth control, defense against phages, biofilm formation, persistence, programmed cell death and general stress response (Unterholzner et al., 2013; Wen et al., 2014). In the present study, either toxin HicA or antitoxin HicB of the *hicAB* cassette were identified in several prophages. Its biological role in *P. larvae* still needs further elucidation, but the *hicAB* cassette significantly influences the mRNA translation process in other bacteria with proposed functions including persister cell formation and involvement in extra cytoplasmic stress responses (Butt et al., 2014; Li et al., 2016; Thomet et al., 2019). The presence of *hicAB* in bacterial genomes has also been associated with HGT (Butt et al., 2014; Li et al., 2016; Thomet et al., 2019). In several prophages, CDS for the antitoxin protein of a TA system were identified, namely the antitoxin part of MazEF and SocAB. MazE antitoxin is the inhibitor of MazF toxin that cleaves mRNA resulting in cellular growth arrest (Simanshu et al., 2013). SocA antitoxin acts as a proteolytic adapter promoting the disruption of SocB inhibiting DNA replication (Aakre et al., 2013). It can be speculated that phages harbor these antitoxins as a defense mechanism, in order to avoid host self-regulation by degradation, not as an added value to their lysogens.

In addition to TA systems, toxins that could affecting the bee larvae were also found in our analysis in prophages, which may can impact *P. larvae* virulence. The EtxB is a toxin that cause enterotoxemia in ruminants and hemolysis in human cell lines (Xin and Wang, 2019) and the sub-unit LukF-PV of the PVL toxin is responsible for the polymerization F component interspersing with S component LukS-PV to form a pore in the target host cell (Spaan et al., 2017), both are pore-forming toxins that among other features are involved in tissue necrosis. The first, the EtxB toxin, has previously been associated with *Clostridium perfringens* (Popoff, 2011; Xin and Wang, 2019) and the latter, the PVL toxin, has been found in prophages in lysogenic *S. aureus* strains (Diene et al., 2017; Coombs et al., 2020). These genes might have been transferred from such bacteria to *P. larvae* by HGT, hypothesis supported by Djukic et al. (2014) and based on the observation of other toxins shares similarities in the different species (Ebeling et al., 2021).

Other prophage CDS seem to influence and increase of AFB severity because might be involved in functions with some effect on larvae tissues. A *P. larvae* infection starts with the bacteria proliferating in the larval gut before it breaches the epithelial layer and invades the hemocoel (Yue et al., 2008). The epithelial layer is lined with a peritrophic membrane consisting of chitins and glycoproteins (Konno and Mitsuhashi, 2019). The degradation of the peritrophic membrane has been shown to be a crucial part of the AFB pathogenesis as it allows direct contact with the epithelial layer and the degraded chitin may serve as a carbon source for *P. larvae* (Garcia-Gonzalez and Genersch, 2013; Garcia-Gonzalez et al., 2014; Ebeling et al., 2016). In this study, CDS for chitin-binding proteins and other proteins that may be involved in the degradation of the peritrophic membrane were identified. One of the prophages, may encode the epithelial and chitin-binding protein GbpA, previously identified in *V. cholerae*, as mediator of bacterial adhesion to human intestinal cells (Kirn et al., 2005). If we assume an analogy with bees, this might confer an advantage *P. larvae*, increase virulence of the host strain by improving bacterial colonization in the larval intestine. In another prophage, enhancin-homologous proteins, belonging to the metallopeptidase family, were observed. Originally described for viruses, enhancin is known to promote infections by degrading the peritrophic membrane of the insect gut. However, enhancin-like proteins has also been found in bacteria, including *P. larvae* (Slavicek, 2012; Djukic et al., 2014) or *Melissococcus plutonius* (Nakamura et al., 2021). In the latter, the causative agent of the honeybee brood disease European Foulbrood (EFB), it is also involved in the degradation of the peritrophic membrane (Nakamura et al., 2021). Another group of proteins from this study belong to the YopX family. Usually associated with pathogenicity, by acting as chaperones for other proteins, they also modulate host cell signaling responses through the type III secretion system (TTSS). Such proteins have also been reported for *Staphylococcus epidermidis* (Gutiérrez et al., 2012) and *S. aureus* prophages (Diene et al., 2017), and for *Lactobacillus plantarum* virulent phages (Kyrkou et al., 2019).

The association between intact prophage CDS and a specific ERIC genotype was evaluated, even recognizing the low number of fully sequenced *P. larvae* genomes in GenBank some conjectures have been formulated regarding their connection with ERIC-genotypes. ERIC I-type strains were the less virulent strains to larvae (Rauch et al., 2009) but also with more genomes available, hold more phage-origin exclusive CDS involved in metabolism (e.g., histidine kinase-like protein, pyruvate dehydrogenase E1, dCMP deaminase family protein, efflux transporter, etc.) than virulence [enhancin and closticin (an antibacterial peptide that inhibits the growth of other bacteria); Kemperman et al., 2003; Table 2; Supplementary Table S5]. The higher frequency of these prophage CDS might be related to the high prevalence of this genotype in AFB outbreaks (Tsourkas, 2020), increasing the opportunity for prophage exchange and acquisition of new genes by HGT. The analysis also revealed that CDS exclusively identified in prophages from ERIC II–V should be able to affect the fitness and virulence. ERIC II strains had proteins with the function of sporulation, membrane transporters, DNA replication and DNA mismatch repair, ERIC III through PgpA participates into the glycerophospholipid metabolism and ERIC IV had proteins related to iron uptake. Prophages from the ERIC V strain, known as a fast larvae killer (Beims et al., 2020), potentially contribute to such trait, encoding virulence genes like leukocidin subunit LukF-PV and GbpA.

Overall, despite the identification of exclusive CDS of all ERIC genotypes, it is not clear whether the CDS involved in virulence are influencing the course of the larval infection. Nevertheless, one exception that seems evident is the presence of toxins in the ERIC V strain analyzed. The remaining exclusive CDS seem to be related with fitness or contributing to *P. larvae* competition with other bacteria.

Yost et al. (2016) earlier suggested that phages displayed host preference for the ERIC group from which they were isolated, and therefore, the possibility of prophages to influence a given genotype was explored, even recognizing the low number of fully sequenced *P. larvae* genomes in GenBank.

Prophages seem to be stable, specific, and important for their ERIC-genotype strains, which allows to infer that the infection of different ERIC-type strains by the same phage is unlikely. This behavior will prevent the occurrence of HGT among the different virulent genotypes.

Within the same host, prophages usually share few similarities, as observed in *B. thuringiensis* (Fu et al., 2019). However, as expected and previously observed in staphylococci phage analyses (Oliveira et al., 2019), the intact prophages have high identity within the same cluster, being all morphologically similar. Furthermore, each cluster harbor intact prophages from the same ERIC or from the closest ERIC genotypes (ERIC I and II or ERIC III and IV strains; Papić et al., 2021). This supports the predisposition of prophages to infect *P. larvae* from the same ERIC genotype from which they were previously isolated, as previously suggested (Yost et al., 2016).

## Conclusion

Overall, this study identified new intact prophages present in all *P. larvae* strains sequenced so far and explored their genomes concerning the potential impact on host strains. Despite some limitations of the *in silico* tools to predict and re-size prophages and the low representativeness on *P. larvae* strains diversity, we introduced important knowledge to the study of *P. larvae* phages by increasing the number of prophage genomes available and annotated.

Moreover, even if phage CDS function was not experimentally confirmed, their diversity in *P. larvae* genomes gave relevant insights on the role of prophages in such pathogen, as relevant matches were found in the database.

## Data availability statement

The original contributions presented in the study are included in the article/supplementary material; further inquiries can be directed to the corresponding authors.

## Author contributions

HR, LM, and AO conceived the study. HR and LM designed the bioinformatics analysis. HR and AN performed the bioinformatics analysis. HR, AN, and LM analyzed the data. HR wrote the original draft manuscript. AO and LM reviewed and edited the paper with contributions from all other authors. AO was responsible for funding acquisition. All authors contributed to the article and approved the submitted version.

## Funding

This study was supported by the Portuguese Foundation for Science and Technology (FCT) under the scope of the strategic funding of UIDB/04469/2020 unit. HR was supported by FCT through the grant SFRH/BD/128859/2017 and COVID/BD/151856/2021.

## Conflict of interest

The authors declare that the research was conducted in the absence of any commercial or financial relationships that could be construed as a potential conflict of interest.

## Publisher’s note

All claims expressed in this article are solely those of the authors and do not necessarily represent those of their affiliated organizations, or those of the publisher, the editors and the reviewers. Any product that may be evaluated in this article, or claim that may be made by its manufacturer, is not guaranteed or endorsed by the publisher.

## Footnotes

^1^ http://phaster.ca/

^2^ https://card.mcmaster.ca/analyze/rgi

## Supplementary materials

The Supplementary materials for this article can be found online at: https://www.frontiersin.org/articles/10.3389/fmicb.2022.903861/full#supplementary-material
